# Supplementary material for: Robotic-OCT guided inspection and microsurgery of monolithic storage devices
Source: Nat Commun. 2023 Sep 14;14:5701. doi: 10.1038/s41467-023-41498-x (PMC10502073; doi:10.1038/s41467-023-41498-x)
Supplement: Supplementary file 1 — Supplementary Information [file 41467_2023_41498_MOESM1_ESM.pdf]

Supplementary Information

Robotic-OCT guided inspection and microsurgery of monolithic storage devices

Bin He[1,2,#], Yuxin Zhang[1,2,#], Lu Zhao[1,#], Zhenwen Sun[1], Xiyuan Hu[3], Yanrong Kang[1], Lei Wang[1], Zhihui Li[1], Wei Huang[1], Zhigang Li[1], Guidong Xing[1], Feng Hua[1], Chengming Wang[2], Ping Xue[2,*] & Ning Zhang[1,*]

[1]Institute of Forensic Science, Ministry of Public Security, Beijing, 100038, China

[2]State Key Laboratory of Low-dimensional Quantum Physics and Department of Physics, Tsinghua University and Beijing Advanced Innovation Center for Structural Biology, Beijing, 100084, China

[3]School of Computer Science and Engineering, Nanjing University of Science and Technology, Nanjing, 210094, China.

* Corresponding author: Ping Xue email: xuep@tsinghua.edu.cn;

Ning Zhang email: zhangning@cifs.gov.cn

[#] These authors contributed equally.

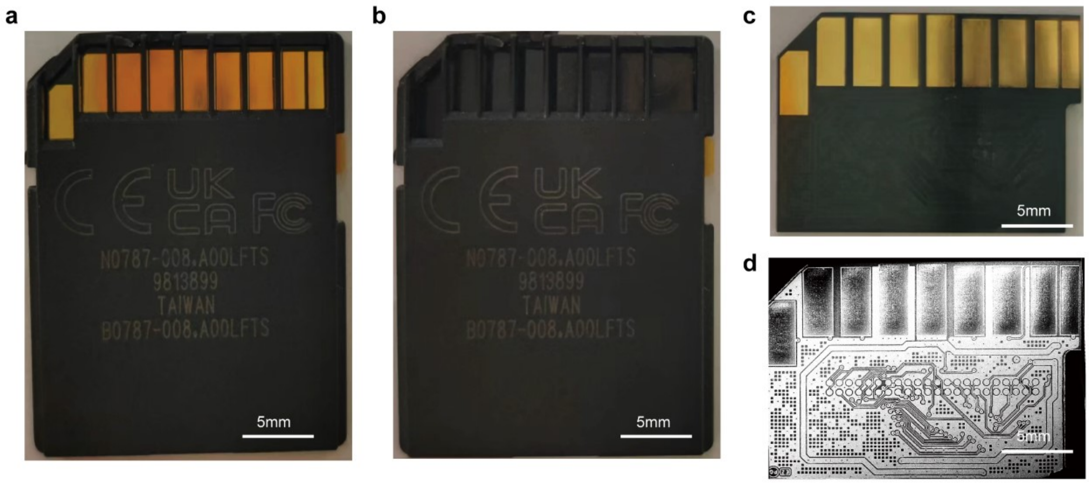

Supplementary Figure 1. Robotic-OCT inspection of an SD Card. (a) Microscope image of the SD card. (b) Outer plastic casing of the SD card that has been removed. (c) The MSD retrieved from the SD card. (d) En face OCT image showing the internal PCB traces of the MSD on the pinout side.

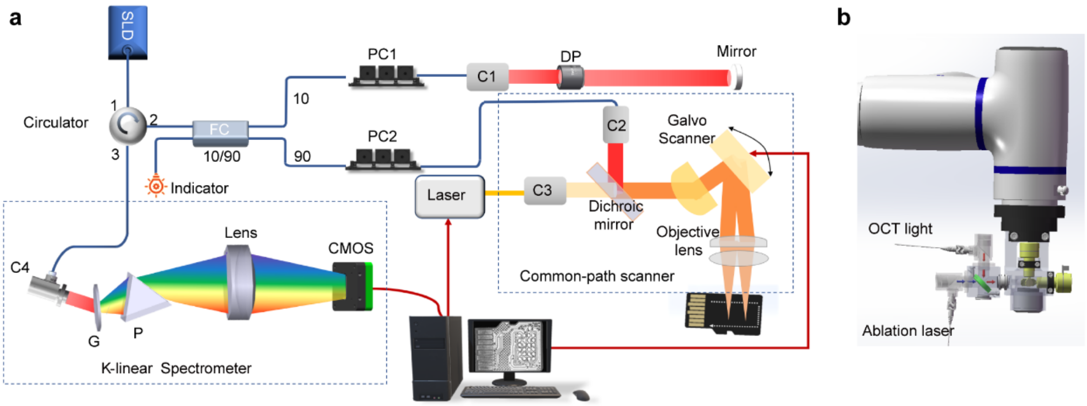

Supplementary Figure 2. Laser ablation microsurgery with robotic-OCT guidance. (a) The schematic of the robotic-OCT-guided laser ablation microsurgery system. (b) A common-path robotic-OCT scanner with the ablation laser.

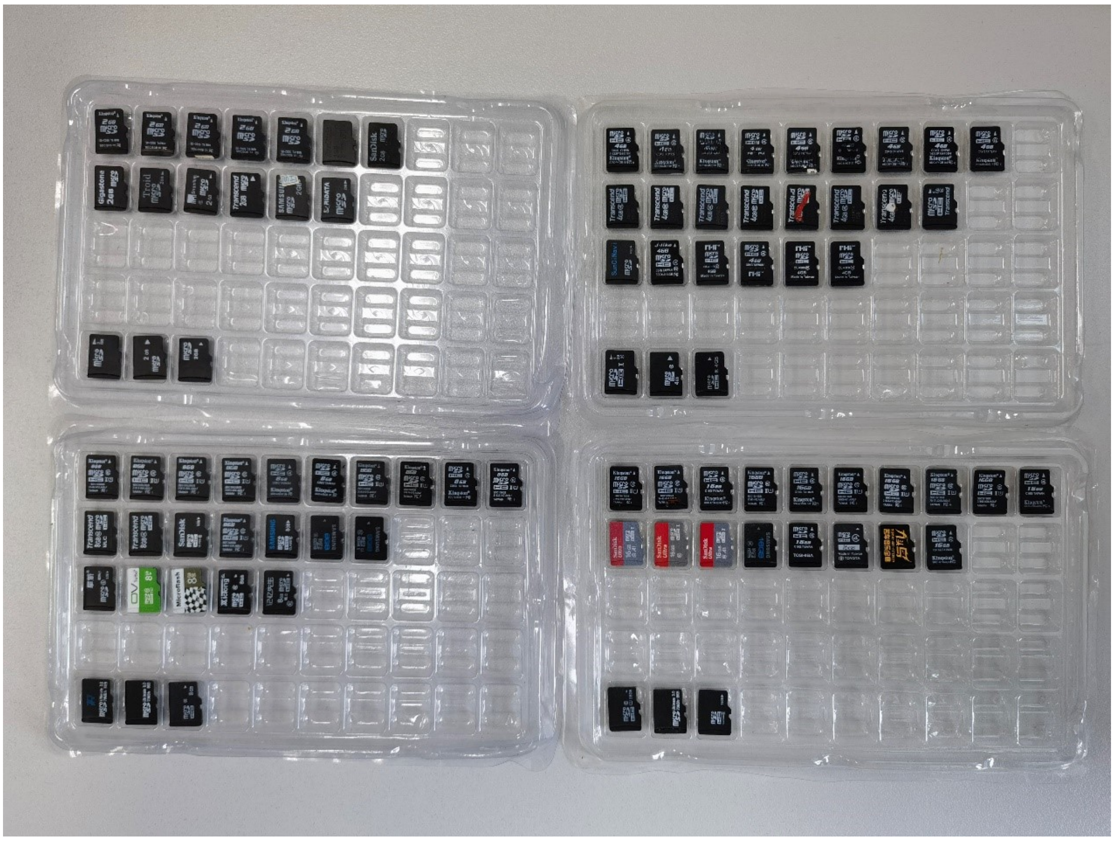

Supplementary Figure 3. Photograph of the micro-SD card dataset used to assess the scattering effects of the insulating layers, including different brands and models collected from multiple sources.

Supplementary Table 1 The MSDs used in our work

Samples	Brand	Capacity	Pinout side surface/Type	Source
Card 1	BanQ	64GB	Matte	Brand-new
Card 2	Kingston	16GB	Glossy	Second-hand
Card 3	Kingston	16GB	Matte	Second-hand
Card 4	Unknown	16GB	Matte	Real case
Card 5	SanDisk	16GB	Scratched	Artificially damage
Card 6	Kingston	2GB	Cracked	Artificially damage
Card 7	Toshiba	16GB	Burned	Artificially damage
Card 8	Kingston	32GB	Matt	Evaluation of laser ablation
Card 9	SanDisk	32GB	Matt	Laser microsurgery
Card 10	ECOJOY	16GB	monolithic USB 1	Brand-new
Card 11	ECOJOY	16GB	monolithic USB 2	Brand-new
Card 12	Transcend	1GB	MMC	Brand-new
